# Advancing research on compound weather and climate events via large ensemble model simulations

**DOI:** 10.1038/s41467-023-37847-5

**Published:** 2023-04-14

**Authors:** Emanuele Bevacqua, Laura Suarez-Gutierrez, Aglaé Jézéquel, Flavio Lehner, Mathieu Vrac, Pascal Yiou, Jakob Zscheischler

**Affiliations:** 1 Department of Computational Hydrosystems, Helmholtz Centre for Environmental Research—UFZ, Leipzig, Germany; 2 Max Planck Institute for Meteorology, Hamburg, Germany; 3 Institute for Atmospheric and Climate Science, ETH Zurich, Zurich, Switzerland; 4 Institut Pierre-Simon Laplace, CNRS, Paris, France; 5 LMD/IPSL, ENS, Université PSL, École Polytechnique, Institut Polytechnique de Paris, Sorbonne Université, CNRS, Ecole des Ponts, Marne-la-Vallée, France; 6 Department of Earth and Atmospheric Sciences, Cornell University, Ithaca, NY USA; 7 Climate and Global Dynamics Laboratory, National Center for Atmospheric Research, Boulder, CO USA; 8 Polar Bears International, Bozeman, MT USA; 9 Laboratoire des Sciences du Climat et de l’Environnement, IPSL, CEA-CNRS-UVSQ, Université Paris-Saclay, Gif-sur-Yvette, France

**Keywords:** Projection and prediction, Environmental sciences, Natural hazards

## Abstract

Societally relevant weather impacts typically result from compound events, which are rare combinations of weather and climate drivers. Focussing on four event types arising from different combinations of climate variables across space and time, here we illustrate that robust analyses of compound events — such as frequency and uncertainty analysis under present-day and future conditions, event attribution to climate change, and exploration of low-probability-high-impact events — require data with very large sample size. In particular, the required sample is much larger than that needed for analyses of univariate extremes. We demonstrate that Single Model Initial-condition Large Ensemble (SMILE) simulations from multiple climate models, which provide hundreds to thousands of years of weather conditions, are crucial for advancing our assessments of compound events and constructing robust model projections. Combining SMILEs with an improved physical understanding of compound events will ultimately provide practitioners and stakeholders with the best available information on climate risks.

## Abstract (web summary)

The authors show that robust analyses of high-impact compound weather and climate events require many samples. Thus, they argue that large ensemble climate model simulations should be used to provide the best available information on climate risks.

## Introduction

Most environmental impacts result from combinations of multiple weather and climate drivers that are referred to as compound events^1–3^. Given the complexity of the climate system, such events can occur in multiple ways, for example involving extreme conditions in different variables happening simultaneously or across space and time^4^. For instance, in 2014/2015, Rio de Janeiro (Brazil) was hit by concurrent extremely hot and dry summer conditions, which led to wide-ranging socio-economic repercussions that exceeded the impacts that would have been caused by heat and dryness in isolation^5^. The hot-dry weather favoured widespread wildfires as well as water scarcity that impacted coffee production^5^. Furthermore, excess fatalities due to a severe dengue fever outbreak were linked to the increase in water storage tanks installed by the population to mitigate the drought^5^. In a similar fashion, impacts from hurricanes typically arise from concurrent hazards via precipitation-driven flooding and wind-driven hazards such as storm surges, as happened when Hurricane Ida led to destruction in Louisiana (United States) in 2021, causing about 30 fatalities^6^. Extreme impacts can also arise from sequences of weather events that increase system vulnerability or lead to disproportionate damages, as exemplified by ecosystem^7^ and agricultural^8^ impacts from the two European drought years in 2018 and 2019, and by the critically reduced water storages in South Africa due to a sequence of three dry winters in 2015-2017^9,10^. Furthermore, combinations of extremes occurring simultaneously (or almost simultaneously) across multiple regions can cause extreme impacts to connected global systems^11^. For example, simultaneous soil moisture droughts across multiple breadbaskets can reduce global food production and threaten food security^12–15^.

Compound events are thus characterized by complex multivariate relationships. Recognizing that a univariate perspective on hazards may severely underestimate risk^16^ led to a rapidly growing body of literature on different types of compound events^3,4,17^, including literature on interconnected, interacting, and cascading risks^18^. However, the limited temporal length of observations and routinely used model simulations often limits the possibility of identifying and studying the sparse multidimensional constellations of climate variables constituting compound events, an issue related to the curse of dimensionality^1,19–21^. Combining routinely used single-member simulations of multiple climate models, such as the standard multi-model ensembles from the coupled model intercomparison project (CMIP)^22^, offers larger sample sizes but leads to confounding irreducible uncertainties arising from internal climate variability (the inherent chaotic nature of climate) and from potentially reducible uncertainties arising from structural differences across models^23–26^. Robustly distinguishing between these uncertainties is important, however, as it can ultimately help reduce uncertainties from structural model differences^25,26^.

In this perspective, we argue that using output from climate Single Model Initial-condition Large Ensembles^24^ (SMILEs) should become standard for studying and projecting compound events. A SMILE consists of many simulations (i.e. ensemble members) from a single climate model based on the same model physics and under the same external forcings, but each starting from slightly different initial states. Thus, each realization in the ensemble evolves differently solely due to internal climate variability. Combining the multiple members of a SMILE ensures therefore a much better sampling of the data sparse regions associated with compound events compared to traditional model simulations^27^. Using a combination of different SMILEs then allows for identifying model differences.

Here we propose six important topics in compound event research and illustrate associated challenges. Each of the following six sections provides information on the importance of one of the six topics and demonstrates, based on our analyses and the literature, both the challenge of addressing the topic and how using SMILEs can be of help in this context. Specifically, we demonstrate that SMILEs are important to (i) robustly assess compound event frequencies and associated uncertainties; (ii) quantify the extent to which anthropogenic climate change has contributed to observed compound events; (iii) provide robust quantification of historical and future changes in compound events; (iv) quantify and explore uncertainties in future projections, including distinguishing between uncertainties arising from internal climate variability and structural model differences; (v) identify most dangerous low-likelihood compound events, advancing their physical understanding, and exploring associated risks; and (vi) evaluate the skill of advanced statistical models developed to improve the assessments of compound events. Via analyses related to four different compound event types, we illustrate how these topics can be addressed using SMILEs. The considered compound event types are compound hot-dry warm-season events, concurrent precipitation and wind extremes, multi-year soil moisture droughts, and concurrent soil moisture droughts across global soybean breadbaskets. See Table 1 for a comprehensive list of the six research topics and the associated analyses related to different compound events.

**Table 1:** The six research topics and the associated analyses related to different types of compound events

Research topic	Analysis	Type of compound event	Data	Figure
(i) Robustly assess compound event frequencies and associated uncertainties	Uncertainty in the historical frequency of compound events	Compound hot-dry event (focus on Southern Asia)	MPI-GE (100 ensemble members)	1
-	-	Concurrent precipitation and wind extremes (focus on Portugal)	MPI-GE (100)	-
-	-	Multi-year drought (focus on Central North America)	CESM1-CAM5 (40)	-
-	Dependence of the uncertainty in the frequency of compound and unviariate events on the sample size	Compound hot-dry event (global scale)	MPI-GE (100)	2
-	-	Concurrent precipitation and wind extremes (global scale)	MPI-GE (100)	-
-	-	Multi-year drought (global scale)	CESM1-CAM5 (40)	-
(ii) Quantify the extent to which anthropogenic climate change has contributed to observed compound events	Required sample size for attribution of compound events to anthropogenic climate change	Generic bivariate compound event	Synthetic data from bivariate normal distribution	3
(iii) Provide robust quantification of historical and future changes in compound events	Effect of internal climate variability on changes in dependencies	Dependence between precipitation and temperature (global scale, with focus on Central Europe)	MPI-GE (100)	4
-	Effect of internal climate variability on changes in compound event frequencies	Simultaneous soil moisture drought over main soybean regions worldwide	MPI-GE (100)	5
(iv) Quantify and explore uncertainties in future projections, including distinguishing between uncertainties arising from internal climate variability and structural model differences	Partitioning uncertainty in the future frequency of compound events (internal climate variability *vs* model differences)	Compound hot-dry event (global scale)	CESM1-CAM5 (40), CSIRO-Mk3-6-0 (30), CanESM2 (50), EC-EARTH (16), GFDL-CM3 (20), GFDL-ESM2M (30), and MPI-GE (100)	6
-	High- and low-risk climate storylines	Compound hot-dry event (South Africa)	-	-
(v) Identify most dangerous low-likelihood compound events, advancing their physical understanding, and exploring associated risks	Identification of extreme event-based storylines	Concurrent precipitation and wind extremes (Portugal)	CESM1-CAM5 (40)	7
-	-	Simultaneous soil moisture drought over main soybean regions worldwide	MPI-GE (100)	-
(vi) Evaluate the skill of advanced statistical models developed to improve the assessments of compound events	Evaluation of statistical models via a perfect-model approach	Simultaneous soil moisture drought over main soybean regions worldwide	MPI-GE (100)	8

## Results

### Estimating compound event likelihoods and associated uncertainties

**Fig. 1:**
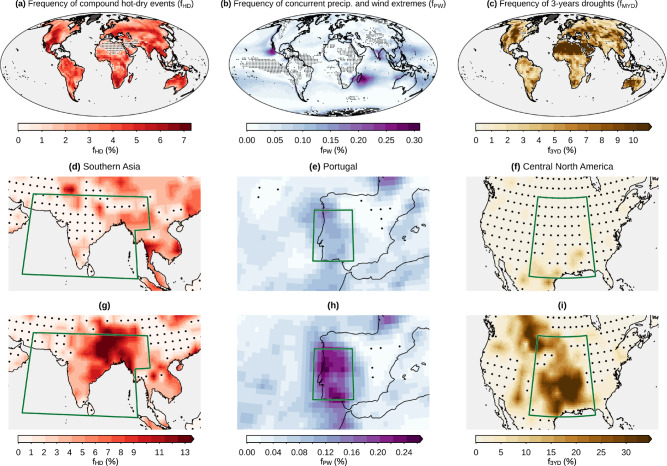
Historical (1950–1980) frequency of compound events and associated uncertainties. **a** Ensemble mean frequency of compound hot-dry events (*f*_HD_; during the warm season) based on the MPI-GE model. Stippling indicates areas where the compound event ensemble-mean frequency is smaller than expected in a reference case that assumes independence between the compound drivers (here, independence between average temperature and precipitation during the warm season). **d**, **g** *f*_HD_ of the ensemble members associated with the 5th and 95th percentiles amongst all the members, respectively, of the *f*_HD_ averaged over the region in the green box. **b**, **e**, **h** As in panels **a**, **d**, **g**, but for concurrent daily precipitation and wind extremes (*f*_PW_) and based on the CESM1-CAM5 model. **c**, **f**, **i** As in panels **a**, **d**, **g**, but for the frequency of three consecutive annual soil moisture droughts (*f*_3YD_). Univariate extremes were defined via percentile-based thresholds, i.e. hot and dry seasons occurring every 10 years on average, precipitation and wind events occurring twice a year, and soil moisture droughts every 5 years (see Methods).

An important challenge of climate studies is assessing the likelihood of extreme events^26^. This information ultimately serves decision-makers, international development agencies, and insurances to develop preparedness for climate impacts^3^. Employing the approach of Bevacqua et al.^26^ and focussing on the historical (1950–1980) frequencies of compound hot-dry warm seasons (*f*_HD_), concurrent daily precipitation and wind extremes (*f*_PW_), and multi-year (i.e., consecutive annual) droughts (*f*_MYD_), we illustrate that using a large sample size is essential for robustly estimating compound event probabilities. Using 3100 years of data from 31-year periods of the 100 ensemble members of the MPI-GE climate model (40 members of the CESM1-CAM5 model for *f*_PW_) allows for robustly identifying hotspot areas where compound event frequencies are higher than what would be expected when assuming that the compound drivers are statistically independent (see unstippled regions in Fig. 1a–c).

Importantly, if such frequencies were estimated based on much smaller sample sizes contained in typically available model simulations and observations, they would be highly uncertain and could ultimately lead to misleading risk estimates. For example, based on 31 years of data from a single ensemble member, someone interested in compound hot-dry event risk over Southern Asia could obtain regionally-averaged *f*_HD_ ranging from 2% to 6% (centred 90% range across the ensemble members), i.e. differing by a factor of 3 (Fig. 1d, g; note that frequencies at the local scale can even range from about 0% to 10%). That means, depending on the data sample selected at random, a wide range of possible risks may be estimated. Similarly, *f*_PW_ estimates could differ by a factor of 2 on average over Portugal, while *f*_MYD_ by a factor of 12 over Central North America. Hence, based on a short sample of data, in principle, a low compound event risk may be estimated over regions that are, instead, at high risk. This sampling uncertainty stems from the fact that 31-year periods are, in this case, highly insufficient to sample internal climate variability, resulting in a wide range of possible frequency estimates across single ensemble members^26^. Similarly, this sampling uncertainty is also inherent in estimates derived from short observational records. SMILEs allow users to obtain a robust model-based estimate of the compound event probability (i.e., the frequency computed from many years of data obtained by pooling multiple ensemble members) and allow for a quantification of how uncertain compound event frequency estimates are based on a given sample size. The latter can be estimated as the range of frequencies across single ensemble members. The differences in compound event frequencies across different ensemble members are likely to partially arise from fluctuations in climate modes of variability that influence regional weather over decadal scales. For example, Atlantic Multidecadal Variability modulates hot and dry conditions in Southern Asia^28^ and droughts in Central North America^29^. SMILE-based analyses can help to assess the influence of the modes of variability on the compound events^12^.

**Fig. 2:**
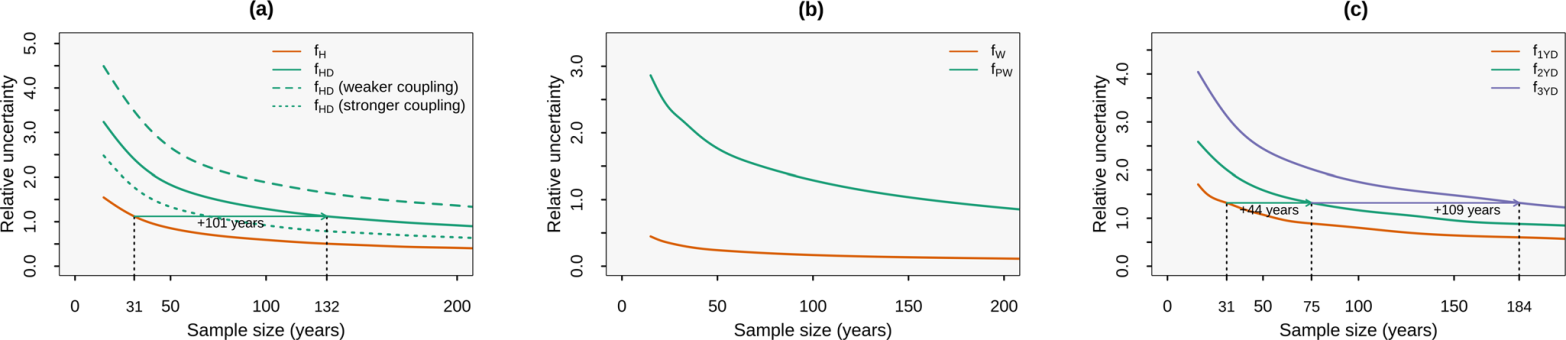
Dependence of the uncertainty due to internal climate variability on the sample size. **a** Relative uncertainty due to internal climate variability in the frequency of univariate hot events *f*_H_ (orange) and compound hot-dry events *f*_HD_ (solid green). Dashed and dotted green lines show the relative uncertainty in *f*_HD_ for grid points with the 35% strongest and weakest coupling, respectively (see Methods). Relative uncertainty is defined as the ratio of twice the standard deviation to the mean of the frequency *f* across ensemble members averaged over global landmasses (see Methods). **b** As in **a**, but for the frequency of wind extremes (*f*_W_, orange) and concurrent precipitation and wind extremes (*f*_PW_, green). **c** As in **a**, but for the frequency of a single-year soil moisture drought (*f*_1YD_, orange), two (*f*_2YD_, green) and three (*f*_3YD_, purple) consecutive annual soil moisture droughts, respectively. The climate models and the definitions of the extremes are the same as in Fig. 1.

It is important to note that the uncertainties in compound event estimates are substantially larger than for univariate extremes independently of the considered sample size, highlighting that employing a large sample size is much more important for compound than for univariate events. This is exemplified by the relative uncertainty in the compound frequency, which is defined here as the ratio of twice the standard deviation to the mean of the frequency across ensemble members^26^. Based on 31 years of data, this relative uncertainty is 2.4 and 1.1 on average over land for compound hot-dry events and univariate hot events, respectively (see continuous lines in Fig. 2a). To reach a relative uncertainty of 1.1 for compound events, 132 years of data would be required (see arrow in Fig. 2a). The results further highlight that the relative uncertainty in the compound frequency is higher at locations where the coupling between the compound drivers is weaker (compare dashed and dotted lines in Fig. 2a), that is, when the statistical dependencies make the compound event rarer, therefore more difficult to sample.

The larger uncertainties for compound than for univariate events holds for very different event types, including compound precipitation and wind extremes (Fig. 2b), despite the fact that the daily scale of precipitation-wind events yields a much larger sample size (compared to compound hot-dry warm-season events). In line with the curse of dimensionality, when we consider multi-year droughts, the relative uncertainties increase with the dimensionality of the considered compound event (Fig. 2c). Hence, employing large sample sizes is even more important for estimating the occurrence of higher-dimensional compound events such as multi-year floods, co-occurring fire drivers^30,31^, concurrent droughts in multiple regions^13^, and spatially extended floods^32^. While arguably our examples are somewhat extreme by focusing on a sample size as small as 31 years, the issues remain for larger sample sizes (Fig. 2). Furthermore, we note that it is not uncommon in the literature that probabilities of compound events are estimated based on such small sample sizes^33–38^, including probabilities of high-dimensional events^13,20,30,39^. Shorter periods than 31 years are often used for projections at fixed global warming levels, e.g. in reports of the Intergovernmental Panel on Climate Change^3^.

### Compound event attribution

In the aftermath of a high-impact weather event, the extent to which anthropogenic climate change has contributed to the event is regularly questioned^40^. Addressing this attribution question is important to gain a better understanding of how climate change has affected and might affect extreme events and their impacts^41^. One way to approach the question is to frame it in terms of a probability ratio PR, defined as the ratio of the probability of an extreme event occurring under current conditions (factual world) to the probability that the event occurs in a world without anthropogenic climate change (counterfactual world)^42^. Such probabilities can be estimated using climate model simulations, for example by comparing runs simulating both worlds^43^, or by using approaches based on non-stationary extreme value theory, where the occurrence of extreme events is fitted against a covariate such as global mean temperature^44^. Values of PR  >  1 imply that climate change contributed to a certain event occurrence; for instance, PR  =  2 implies that climate change made a certain event class (typically defined as all events exceeding a critical threshold) two times more likely. So far, attribution studies have mainly treated events as univariate, however—because many climate-related impacts are caused by a combination of multiple drivers—studies on compound event attribution are emerging^43–46^.

As attribution studies typically focus on very extreme events, they are often based on large ensemble climate model simulations. For example, the weather@home experiment, a large ensemble of simulations of the regional atmosphere-only climate model HadRM3P^47^ has been widely used by the attribution community. Other studies have relied on the multi-model CMIP5 and CMIP6 ensembles to gather enough data to isolate any climate change forced signal from internal climate variability noise, though the ability to disentangle internal variability from model differences ultimately depends on the number of runs available for each model^24,48^. The required ensemble size to estimate internal variability, which is needed to provide robust attribution assessments, depends on several factors, such as the spatio-temporal scale of the event, the variable, and the amplitude of the forced trend^49^.

**Fig. 3:**
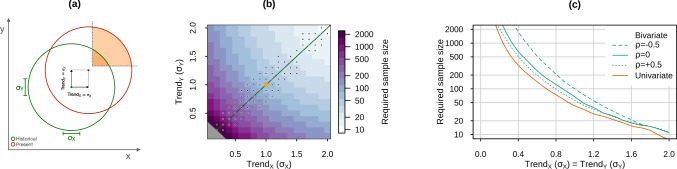
Required sample size for attribution of compound and univariate events. **a** Idealized bivariate distribution of two compound drivers (*X*, *Y*) under historical conditions (green), and under present conditions (red) obtained via shifting the mean of the historical drivers by forced trends Trend_i_ equal to one standard deviation (*σ*_*i*_) of the historical distribution. Shading in the top-right depicts compound events, i.e. concurrent high extremes. **b** Minimum sample size required for attribution of concurrent high extremes (>90th percentile) of synthetic data simulated from a bivariate standard Gaussian distribution (with constant zero correlation) in the presence of positive forced trends Trend_X_ and Trend_Y_ in the two drivers (expressed in units of standard deviations). Grey on the bottom bottom-left indicates a required sample size larger than the values in the palette. The large orange dot indicates the combination of trends displayed in **a**. Stippling indicates combinations of Trend_Y_ and Trend_X_ for which attributing the compound event requires a larger sample size than that needed for attributing any of the underlying univariate events (elsewhere compound attribution requires a sample size in between that needed for the two univariate events in isolation). **c** Sample size required for attributing any of the two univariate events (orange), and the compound event (green) assuming equal forced trends for both drivers and different constant correlation (*ρ*) between the drivers (see legend).

Here, we demonstrate that for compound event attribution, larger sample sizes are required than for univariate event attribution, in particular if the drivers are not strongly correlated and have trends of similar magnitude. We build on the synthetic data approach developed by Zscheischler and Lehner (2022)^43^. For the sake of simplicity, we focus on events that increase in probability with climate change (Fig. 3a). That is, we calculate the required sample size for the attribution of concurrent high extremes of climate variables that show positive trends due to anthropogenic climate change such as coastal flooding followed by a heatwave^50^. We define the required sample size as the number that guarantees correctly identifying PR > 1 in the presence of a forced trend, assuming no trend in the dependence between drivers^43^ (see Methods). In general, very large sample sizes are needed to disentangle small climate change driven trends from internal climate variability (Fig. 3b). We find that when trends in the drivers are comparable, attributing compound events requires a larger sample size than that needed for attributing any of the contributing univariate events (see stippling in Fig. 3b). In particular, a substantially larger sample size for compound than for univariate events is needed for smaller forced trends (Fig. 3c). For example, the sample size needed under identical trends of 0.4 ⋅ *σ*_*i*_ in the drivers differs by about 330 (i.e. by a factor 2) between the bivariate (under zero correlation) and the univariate cases (Fig. 3c). Furthermore, larger sample sizes are required when the dependence between the compound event drivers is small or negative (Fig. 3c), i.e.—in general—when the dependence makes the compound event rare. For example, the sample size needed under identical trends of 0.4 ⋅ *σ*_*i*_ in the drivers differs by about 1200 (a factor 3.5) between the bivariate case under −0.5 and +0.5 correlation (Fig. 3c). When the trends in the compound variables differ strongly in magnitude, attributing the compound event requires a sample size in between what is needed for attributing the univariate events in isolation (missing stippling in Fig. 3b). That is, for example, attributing a compound hot-dry event to anthropogenic warming trends requires a larger sample size than that needed for attributing the heat event in isolation, though smaller than for attributing the drought event alone given that trends in droughts are much weaker than trends in heatwaves in most regions^3^.

### Detecting changes in compound event characteristics

Internal climate variability arising from the chaotic nature of the climate system can obscure anthropogenic climate change signals within short time periods^51^. Hence, especially for low-probability events, robust sampling of internal climate variability under different global warming states is crucial for assessing anthropogenically-forced changes robustly^51^. For compound events, identifying anthropogenically-forced changes requires, in addition to considering changes in the individual compound event drivers, to also quantify changes in their coupling, i.e. their statistical dependence^32,52–54^. However, quantifying any potential anthropogenic signals in the dependence, and therefore understanding the processes driving such signals, has proven to be particularly challenging so far due to the limited sample size of considered datasets^33,34,38,52,54^. For example, for compound coastal flooding—which is often driven by concurrent wind and precipitation extremes^54^—Wahl et al.^52^ identified an increase in the dependencies between the drivers in the historical period over the United States’ coast, but it was not possible to attribute this increase to anthropogenic climate change. Bevacqua et al.^54^ suggested that internal climate variability may dominate such dependency changes even under high greenhouse gas emissions and that SMILEs may therefore be necessary for identifying any potential forced changes. The SMILE experimental design allows for a quantification of the forced response (including changes arising from mean and variability^55^ of individual compound drivers, as well as from their dependence), by averaging changes across ensemble members, hence filtering out spurious trends due to internal climate variability. Once SMILE simulations allow us to disentangle such changes, further analysis can be carried out to identify the physical mechanisms driving these changes.

**Fig. 4:**
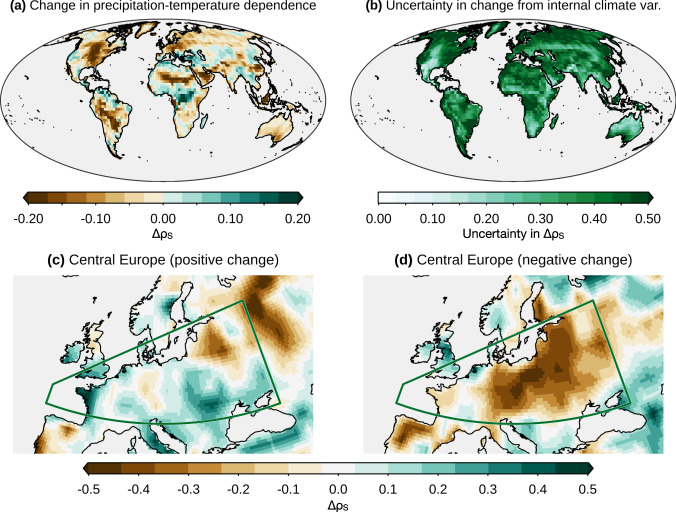
Internal climate variability can obscure forced changes in intervariable dependence. **a** Ensemble mean (MPI-GE model) change in Spearman correlation (Δ*ρ*_*S*_) between summertime mean temperature and mean precipitation in a 2 °C warmer world relative to preindustrial conditions. **b** Uncertainty in Δ*ρ*_*S*_ due to internal climate variability (twice the standard deviation of Δ*ρ*_*S*_ across ensemble members, i.e. 2 ⋅ *U*_IV_; see Methods). **c**, **d** Δ*ρ*_*S*_ of the ensemble members associated with the 5th and 95th percentiles amongst all the members, respectively, ranked by Δ*ρ*_*S*_ averaged over Central Europe.

Focussing on compound hot-dry events, previous studies based on single-member simulations highlighted disagreement among model runs on the sign of the changes in the statistical dependence between temperature and precipitation^53^, ultimately causing large uncertainties in impact assessments. In particular, changes in this dependence are expected to modulate the heat sensitivity of crops as temperatures rise, thereby affecting crop production^56^. We illustrate that such changes in the dependence can be robustly estimated using the large ensemble simulations of the MPI-GE model as an example (Fig. 4a). Highly misleading results in the strength and sign of the changes may be obtained based on short time periods (31 years) from a single model simulation (note that this sample size is similar to that used in previous studies^33–35,38^). This is due to the large uncertainty due to internal climate variability in the dependence change (Fig. 4b, compare with Fig. 4a). Thus, using model output from only one model simulation does not even guarantee to identify the correct sign in the projected change. For example over Central Europe, one may identify either a positive or negative change (Fig. 4c, d) despite the clear underlying negative forced change (Fig. 4a).

**Fig. 5:**
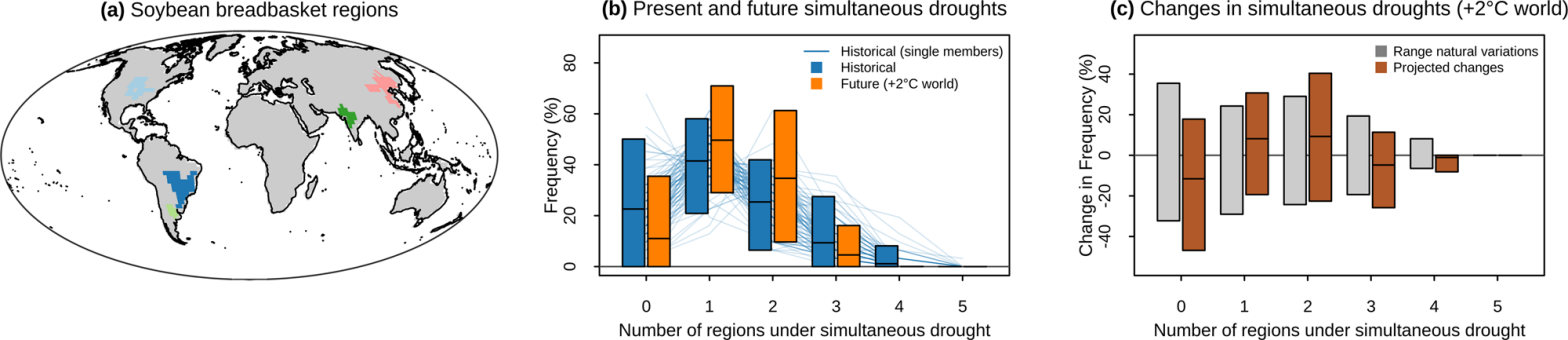
Internal climate variability can obscure forced changes in compound events. **a** The five soybean regions. **b** Historical (blue) and future (orange) frequency of annual soil moisture drought (defined as events occurring every 4 years on average) occurring in *N* simultaneous (*x*-axis) soybean regions (based on MPI-GE model). Coloured bars indicate the centred 95th percentile range and the mean amongst the ensemble members (historical probabilities based on individual members are shown through background lines). **c** Projected change in probabilities of simultaneous droughts in a 2 °C warmer world relative to preindustrial conditions (brown) and variations between two historical periods due to internal climate variability (grey; 95% range of changes obtained from randomly paired historical ensemble members).

Similar considerations apply to the overall changes of compound event occurrence (not just changes in statistical dependence of drivers), as exemplified for trends in spatially compounding events. A previous study, by comparing two recent adjacent 20-year observational periods, indicated an increase in the probability of multiple breadbasket failures for wheat, maize, and soybean, and a decrease for rice^13^. However, model simulations (MPI-GE model) indicate a large variability in the frequency of a similarly defined 5-dimensional compound event, i.e. concurrent annual soil moisture droughts across the five main soybean breadbaskets worldwide (Fig. 5a), when estimated based on just 31 years of data (derived from a single ensemble member) (see blue bars in Fig. 5b). As a result, even two consecutive 31-year periods under the same historical conditions could exhibit a large increase or decrease in this frequency solely due to internal variability (grey bars in Fig. 5c). This indicates the challenge of attributing detected changes in spatially compounding events to anthropogenic climate change without employing large sample sizes. Notably, the potential for detecting both positive and negative changes persists even when considering projections in a world 2 °C warmer than pre-industrial conditions (brown bars in Fig. 5c, and Fig. 5b), in which case forced changes are expected to be much larger than in the historical period. Here, large ensemble simulations allow for robustly identifying a future forced response. For instance, by merging one-hundred ensemble members of 31 years, the change in the probability of *N* concurrent regions under droughts (lines overlaid to brown bars in Fig. 5c), is statistically significant (95% confidence level) up to *N* = 4.

### Uncertainty sources in projections and plausible climate storylines for impact modelling

Quantifying and understanding uncertainties in climate projections and identifying the potentially wide range of plausible climate futures is highly relevant for policymakers^25,57,58^. It is important to distinguish between uncertainty arising from internal climate variability, which is irreducible because of the inherently chaotic nature of the climate system, and uncertainty from structural differences amongst climate models, which could be reduced through, for instance, model improvements or emergent-constraints^59^. Uncertainty partitioning based on multi-model single-ensemble simulations^23^ is expected to be biased, especially at regional scales and for variables with large variability^60^, which includes compound event frequencies. Here, we illustrate how multiple SMILEs can be used to partition uncertainties in compound event projections following recently proposed approaches^25,26^ (see Methods).

**Fig. 6:**
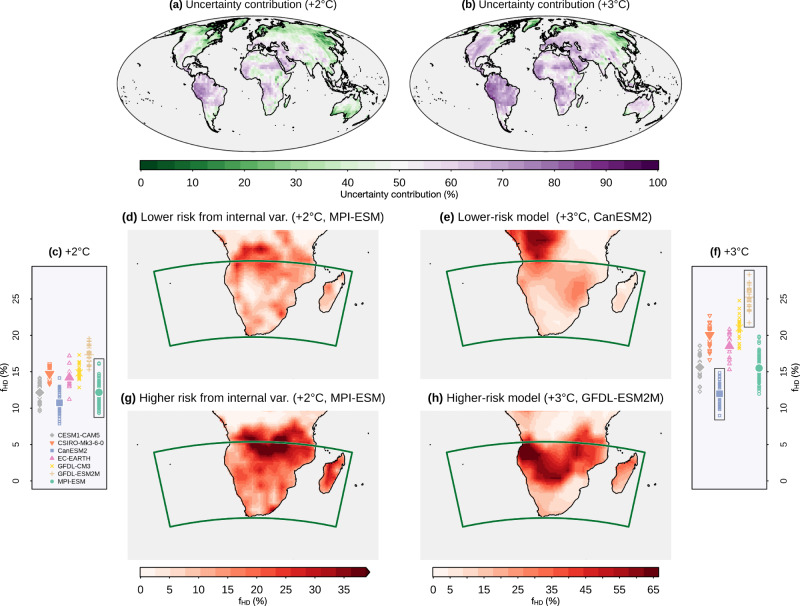
Model differences dominate internal climate variability at higher warming levels. **a**, **b** Uncertainty in the frequency of compound-hot-dry events (*f*_HD_) due to model-to-model differences (*U*_MD_) relative to the sum of *U*_MD_ and the uncertainty due to internal climate variability (*U*_IV_) in a world 2 °C (**a**) and 3 °C (**b**) warmer than pre-industrial conditions (expressed in percentage; uncertainty is dominated by model-to-model differences for values above 50% and by internal climate variability otherwise). **c**, **f** Average of the future *f*_HD_ over Southern Africa (green box in **d**) according to individual ensemble members of different climate models in a 2 °C and 3 °C warmer world, respectively (larger symbols indicate ensemble mean of individual models). **d**, **g** In a 2 °C warmer world, climate storylines of compound hot-dry events resulting from internal climate variability, i.e. future *f*_HD_ from the ensemble members associated with the lowest and highest values averaged over Southern Africa amongst all the members of the MPI-GE model (shown in the box in **c**). **e**, **h** In a 3 °C warmer world, storylines resulting in the lowest and highest risk model, i.e. ensemble mean of the future *f*_HD_ associated with the two models out of seven (shown in the boxes in **f**) showing the lowest and highest values averaged over Southern Africa. Compound hot-dry events were defined as in Fig. 1.

For compound hot-dry events, we demonstrate that contributions from internal climate variability and structural model differences to uncertainties in compound event projections depend on the warming level, with the two sources broadly contributing comparably in a 2 °C warmer world (Fig. 6a) and model differences dominating in a 3 °C warmer world (see purple dominating in Fig. 6b). Uncertainty in climate projections (i.e. in the climate over a period of, for instance, 30 years) can be communicated via *climate storylines*, which are distinct plausible future climates^61^ that can be derived from large ensemble climate model simulations^26,62,63^. For example, one storyline could be derived from a model simulation that exhibits an increase in precipitation over a region of interest, while another from a simulation that exhibits a precipitation decrease. Note that climate storylines may also be used as input to impact models to allow for an efficient exploration of the full range of plausible future climates and associated impacts (see final discussion).

Distinct climate storylines derived from different ensemble members of a single SMILE^26,62^ allow for communicating “certain” irreducible uncertainty stemming from internal climate variability. In fact, the robust sampling of internal variability offered by the large number of ensemble members of a SMILE allows for identifying very extreme, albeit plausible future scenarios that may lead to the largest impacts^26,61,62^. An illustration of climate storylines with different compound hot-dry event frequencies in a 2 °C warmer world over Southern Africa is provided in Fig. 6d, g. When uncertainties from model differences dominate, as it happens in a 3 °C warmer world in this example, climate storylines derived from different models can be used to communicate such uncertainties transparently (Fig. 6e, h). In principle, such uncertainty from model differences could be reduced by improving the representation of the processes driving compound event changes. For example, in Southern Africa, improving the representation of processes dominating precipitation uncertainties, in particular related to shifts in the convective region and the structure of sea surface temperature in the surrounding oceans^64^, is essential to reduce uncertainties in future compound risk^26^.

### Event-based storylines

The largest socioeconomic impacts are often caused by individual very rare events^65,66^. Estimating the occurrence probabilities of such rare events is very challenging, but a better understanding of the events’ dynamics is critical for risk analyses. This can be done using an *event-based storyline*^67^, which represents a physically self-consistent unfolding of a plausible individual event. The concept of event-based storyline differs from that of climate storyline introduced above, as the former refers to a plausible individual event (e.g., a very intense storm or crop failure and its consequences), and the latter to a plausible future climate over a long period (e.g. 30 years). The concept of event-based storylines has been employed to study several types of rare high-impact events over recent years^68–72^. Event-based storylines can be used to improve our understanding of the physical drivers of very extreme events and, through collaborations between climate scientists and impact modellers, the event’s socio-economic consequences^66^. However, identifying rare high-impact events that typically consist of combinations of multiple climatic drivers from short samples of data is difficult^73,74^. For example, based on datasets with limited sample size, identifying and studying the potential for combinations of physical drivers that lead to record-shattering heat events can be difficult^73^, and therefore the threats of such rare but plausible extreme events could be neglected.

In this context, the large sample of atmospheric conditions obtained by pooling multiple ensemble members of a SMILE can be used to identify very rare and potentially high-impact events that are plausible but have not yet occurred in the observational record (so-called unseen events^73,75,76^), which is particularly useful for compound events as we illustrate with two analyses. To quantify rarity in a multi-dimensional space we rely on a copula-based approach^77,78^ by selecting events that maximise the copula describing the distribution of the compound event drivers (see Methods).

**Fig. 7:**
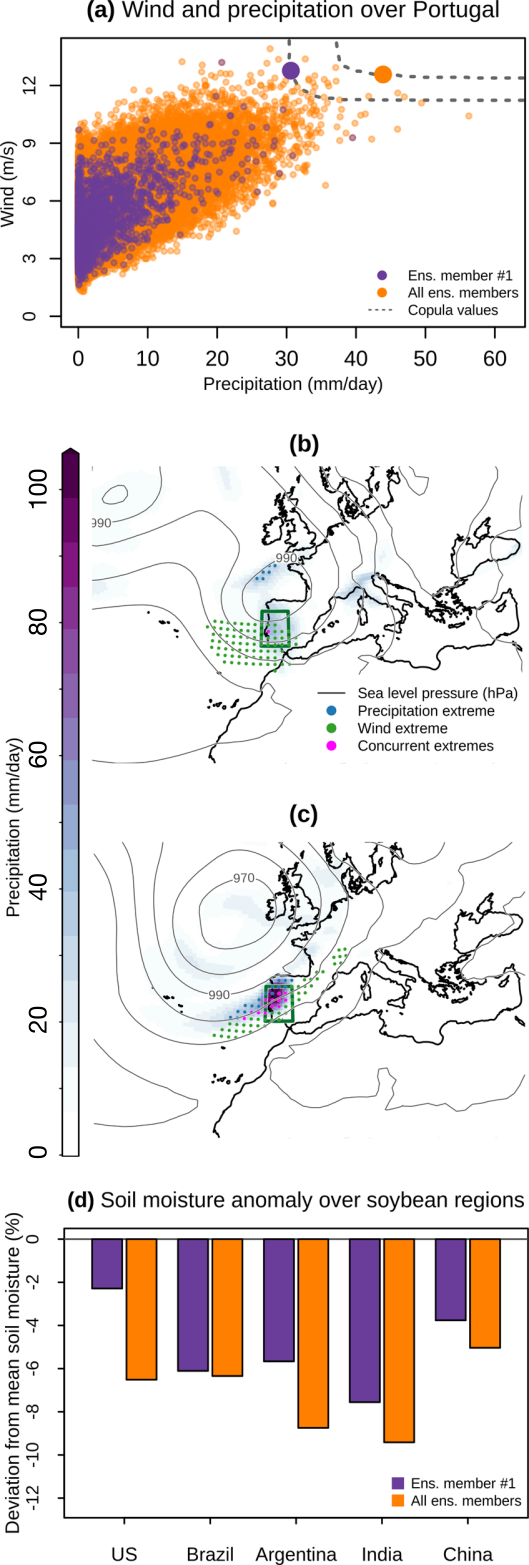
Identifying extreme event-based storylines. **a** Pairs of wintertime (December-February, 1950–1980) daily-mean precipitation and wind values averaged over Portugal (box in **b**, **c**) based on data of the first ensemble member (violet) and based on all pooled ensemble members (orange) of the CESM1-CAM5 model. Large dots indicate the most extreme compound events in the two datasets, i.e. the selected event-based storylines (Methods). **b**, **c** Precipitation (shading) and sea level pressure (isolines) associated with the selected extreme events based on one (**b**) and all ensemble members (**c**). Stippling indicates locations exhibiting extreme precipitation (blue), extreme wind (green), and concurrent extremes (magenta), where extremes are defined as values exceeding the local 10-year return levels—based on December–February data. **d** Anomalies (%, with respect to the mean; 1950–1980) of annual regionally averaged soil moisture over the five soybean breadbasket regions according to the most extreme compound event storyline based on data from the first ensemble member (violet) and based on all pooled ensemble members (orange) of the MPI-GE model.

The first analysis concerns compound precipitation and wind extremes over Portugal. Using all available simulations from multiple ensembles of the model CESM1-CAM5, we find that much more extreme events are possible compared to the most extreme event identified in a single simulation of that model (Fig. 7a). Specifically, based on the first ensemble member we identify as the most extreme event an extratropical cyclone with a central pressure below 990 hPa, which causes wind extremes along the Portuguese coast, but virtually no heavy precipitation (Fig. 7b). Based on all simulations the most extreme event is a deep cyclone with a core pressure below 970 hPa, which causes extreme wind speeds and daily precipitation above 100mm in Portugal (Fig. 7c). Such an event may cause a combination of inland flooding and landslides^2^, coastal storm surges^54^, uprooted trees, and downed power lines^79^. In a second analysis, we search for concurrent droughts in five key soybean production regions using data from the model MPI-GE. The most extreme event based on all pooled ensemble members is associated with droughts that are characterized by (sometimes substantially) lower soil moisture over all regions than based on the first ensemble member alone (Fig. 7d). Here, in line with a recent SMILE-based analysis^12^ that revealed the contribution of oceanic modes of variability on concurrent droughts across multiple breadbasket regions, further analyses could help identify plausible oceanic precursors of worst-case spatially compounding droughts. In general, the simulated worst-case events should be considered plausible only if the driving processes are in line with basic physical principles^80^.

### Developing and evaluating statistical tools for compound events analysis

Properties of compound events are often studied via statistical modelling^2,16,20,81,82^. For example, statistical weather generators, which are used to simulate realistic weather conditions, can—in principle—allow sampling unobserved extreme events and estimating the likelihood of very rare events, reducing issues related to the limited sample size of observations^77^. To meet the challenge of studying different aspects of compound events, new statistical methodologies of different levels of complexity are being developed^2,20,83–86^. However, novel statistical tools need to be tested and evaluated, and the limited sample size of observational records is a constraining factor in this regard.

Building on a *perfect-model approach* (introduced below), SMILEs offer a powerful testbed for new and existing methodologies^24,75^. This can be particularly useful for assessing the skills of novel methods in compound event research, which can be very complex given the need for modelling multiple inter-variable relationships and multivariate extremes^87,88^. In a perfect-model approach, the climate represented by the large ensemble simulations of one climate model is used as a testbed. The statistical model is calibrated to the data of a single ensemble member, representing the pseudo-observations that are limited in sample size. Then, the skill of the statistical model in representing the statistics of interest in the underlying climate can be evaluated against the climate derived from all other ensemble members.

**Fig. 8:**
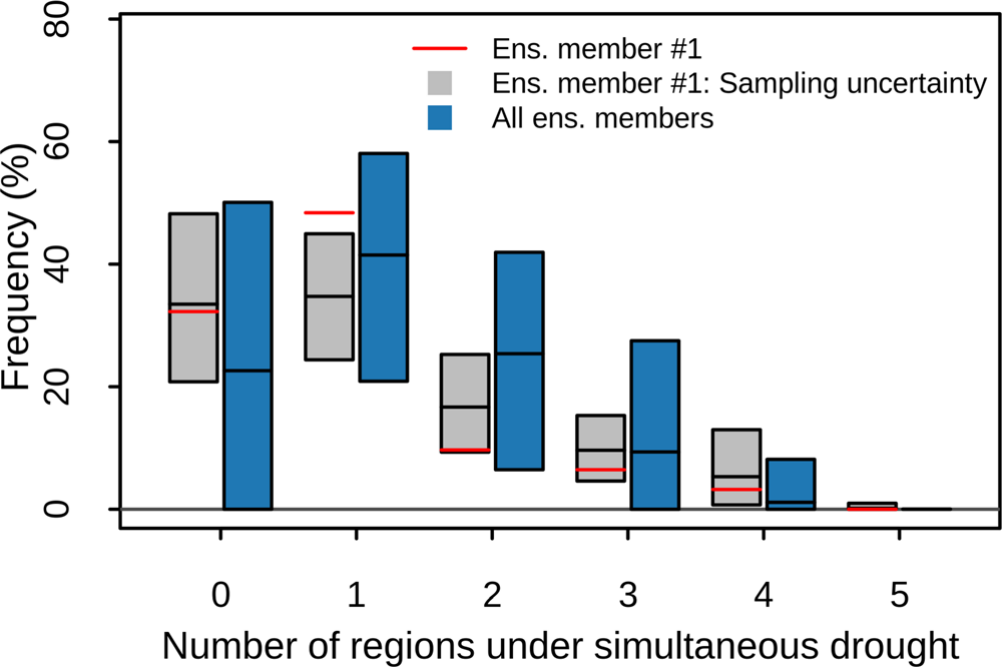
Evaluating statistical modelling of compound events using large ensemble simulations. Blue bars as in Fig. 5b. Red line, the historical probability of annual soil moisture drought in *N* simultaneous regions based on data of the first ensemble member. Grey bar, sampling uncertainty (centred 95% confidence interval) in the historical probability estimated through a statistical model fitted to data of the first ensemble member (the mean prediction of the statistical model is shown by the black line).

For example, one could be interested in evaluating a multivariate statistical model that is fitted to 31 years of soil moisture observations across the five soybean breadbasket regions with the goal to estimate the probability of simultaneous droughts in the five regions. We illustrate using the perfect-model approach (Methods) that a statistical model based on pair-copula-constructions, a statistical tool that is widely used for high-dimensional compound events^13,20,89^, underestimates the probability of two regions under drought in favour of a possible overestimation of four regions under droughts (Fig. 8; the correct probability shown by the black line in the middle of the blue bar is outside of the sampling uncertainty in grey of the statistical model). The statistical model does also not represent the statistics of the data used for calibration (the red lines do not always fall inside the grey range), which in principle may be due to structural biases in the chosen statistical model, indicating the challenge of developing multivariate statistical models for compound events based on limited sample sizes and the advantage offered by the large sample size of SMILEs. While it seems unwise to estimate high-dimensional probabilities from limited sample sizes, this is not uncommon in the literature^13^. Finally, note that the uncertainty in the frequency of concurrent droughts arising from internal climate variability (blue bars; the range of frequencies that can be measured in a given 31-year period) is conceptually different from the sampling uncertainty from the statistical model (grey bars; an uncertainty around the frequency averaged across multiple 31-year periods), hence—based on theory—a match between these uncertainties should not be expected. Finally, we note that a user may employ a perfect model approach to test different types of statistical models and eventually choose the most appropriate one.

## Discussion

While large ensemble simulations are known to be important to study very extreme univariate events^24,73,90,91^, we demonstrated that such simulations are even more relevant for compound event analysis. In principle, when studying mild extremes under stationary climate conditions, pooling data from observations or a historical single ensemble member could increase sample size and partially reduce uncertainties. However, for studying very extreme compound events and non-stationary climate conditions, very large sample sizes from multiple ensemble members are essential^14,26,32,92–95^. Furthermore, climate models are an essential tool for investigating future changes in compound events. We recommend using SMILEs to avoid estimating potentially incorrect compound event frequencies and associated changes. SMILEs are especially important when the correlations between the compound drivers are weak and for very high dimensional compound events, that is, when identifying compound event characteristics becomes particularly difficult based on short sample sizes.

As we illustrated with several analyses, the large sample sizes of SMILEs allow for tackling the challenge of robustly estimating statistical features of compound events. However, we note that—as for virtually all of the research related to climate and weather—research gaps do not arise merely from statistical challenges. It is important to advance our understanding of the physical processes behind compound events and their changes via in-depth analyses that combine models and long observational datasets. The large sample size of SMILEs can help advance the physical understanding of compound events. For example, a SMILE-based analysis revealed that a relatively small percentage increase in precipitation intensities caused by a combination of dynamic and thermodynamic atmospheric changes will drive a disproportionately larger increase in the spatial extent of precipitation extremes^32^. Similarly, SMILEs helped reveal that the concurrence of El Niño with the cold phase of the Tropical North Atlantic can substantially enhance the probability of concurrent droughts over global breadbasket regions^12^. Such climate-model-based analyses should be carried out side by side with a climate model evaluation of the primary processes responsible for compound events (see discussion below), e.g. the climate modes of variability.

SMILEs can also help advance our understanding of very extreme events. For example, climate model simulations helped uncover the physical drivers of record-shattering hot events^73,96^. Given that climate models can have biases, very extreme simulated compound events should be considered plausible only if the identified driving processes are consistent with basic physical processes^80^. Models that are unable to capture events as extreme as those observed even with the improved sampling provided by SMILEs are likely affected by biases in their representation of real-world processes.

SMILEs further allow for robustly quantifying forced changes in compound event features, including the dependencies between compound drivers. In fact, large changes may appear when analysing data with limited sample size, although no forced trend may exist. This effect, which also exists for univariate quantities^51^, is much enhanced for compound events. Once forced changes are identified, they can be linked to their driving physical processes. For example, SMILEs can help disentangle the physical mechanisms behind dependence changes, e.g. it could be studied whether the strengthening of land-atmosphere feedbacks^97^ strengthens temperature-precipitation dependencies^53^, whether projected changes in storm tracks^54,98^ influence the dependence between precipitation and wind extremes, and how changes in dependence scale with global warming.

While SMILEs offer many advantages compared to single-member climate model simulations, a series of considerations are worthwhile. SMILEs have already helped substantially to advance our understanding of climate variability^24^; however, climate models can have biases that should be considered carefully. That means climate model skills need to be evaluated^43,86,99^. Assuming that any difference between model- and observation-based estimates is only due to biases can be misleading as, especially for multivariate relationships, large differences may arise from internal climate variability^43,54^. That is, climate models with a few or even one single member may fail to capture observed behaviours not because of model biases but simply because of insufficient sampling of internal climate variability. The improved sampling of internal climate variability in SMILEs allows for better model evaluation^100^—specifically, it allows for avoiding rejecting models for the wrong reason. In general, we highlight that a purely statistical assessment of the model skills in representing the ultimate quantity of interest (e.g., frequency of extreme events) should always be complemented by a process-based approach that evaluates the model representation of the driving processes of extreme events^72,80,101^ and therefore the physical realism of the simulated events. Here, we argue that the latter, process-based oriented evaluation is particularly relevant for compound events. In fact, when observations are very short in sample size, while SMILE-based model evaluation can avoid rejecting models for the wrong reason, it may be very difficult to disentangle model biases from noise arising from internal climate variability in highly uncertain statistical quantities such as the frequency of compound events.

Models that pass a process-based climate model evaluation may still have residual biases^102^. Hence, bias adjustment or downscaling methods are operationally used for impact assessments^102^. For compound events, multivariate downscaling methods^103^ or bias adjustments^104–107^—which adjust both the distributions of the individual variables of interest and their statistical coupling—should be considered^104,108^. Correcting SMILEs can be challenging^80,104^; however, there are recent developments that provide bias-adjusted large ensembles^80,109^ (e.g., the CanLEADv1^110^), as well as dynamically downscaled SMILEs^27^ (i.e., SMILE from regional climate models that should better represent local processes). Building on a *perfect-model approach*^24,75^, SMILEs could also be used to assess the skill of multivariate bias-adjustment methods^104^.

In general, running impact models for many SMILEs can be challenging due to the computational costs of some impact models and the need of bias adjusting climate models^111^. In this context, storylines derived from SMILEs can be used to explore and communicate a wide range of plausible future climates over a period of, for instance, 30 years (via climate storylines, Fig. 6), as well as the consequences of individual worst-case events (via event-based storylines, Fig. 7). The selection of such storylines should be based on a metric that takes into account the combinations of drivers that cause impacts to a given sector of interest^72^, hence the choice of the metric will depend on the sector and should be guided by impact modellers. For example, exploring impacts associated with a year characterised by widespread droughts over breadbasket regions (event-based storyline) may allow testing different adaptation strategies related to, for instance, irrigation, therefore supporting decision-making^1,2,66,68^, whereas climate storylines derived based on frequencies of cold spells occurring together with low winds might be explored for the renewable energy sector^112^. Overall, employing a limited set of storylines as input in impact models can allow for efficient exploration of a substantial range of possible impacts, therefore supporting climate risk assessments^65,72^. Given the cost of dynamically downscaling full SMILEs, downscaling single events or ensemble members can provide an effective approach for ultimately driving impact models with high-resolution climate data^72^.

In our illustrations, we employed seven CMIP5 generation global SMILEs^24^ (each providing 16-100 ensemble members, see Table 1). However, new generation CMIP6 outputs that include more SMILEs are now becoming available (see https://www.cesm.ucar.edu/projects/community-projects/MMLEA/ for a current overview). The substantial reduction of uncertainties in compound event frequencies with the increase in the sample size (Fig. 2) indicates that the many CMIP6 models that are now available with several ensemble members (16 models with at least 10 members), will offer new opportunities for studying different types of compound events. Considering such a variety of models will enable a more comprehensive study of uncertainties from model-to-model differences in climate risk assessments. Furthermore, new SMILEs from regional climate models have also started to increase in number and can allow for comprehensive regional analyses by combining regional and global SMILEs^27^. The increase in computational resources should eventually enable the generation of high-resolution SMILEs at the global scale. We would like to note that alternatives to SMILEs exist. For example, climate emulators^113,114^ simulate large sample sizes at a low-computational cost; however, despite some exceptions^88^, they are typically not yet tailored to compound events. Furthermore, combining our understanding of physical processes with intelligent use of methods can also help reduce required sample sizes and thus render single-member model simulations more useful. For instance, using approaches termed *dynamical adjustments* allows estimating the forced response of climate change on various quantities of interest under certain assumptions with comparably small sample sizes^115,116^.

Based on a number of illustrative analyses (Table 1), we conclude that assessing compound events and their changes based on datasets of limited sample size may be misleading. Observational data, particularly when not characterised by a very small sample size, remain paramount for analysing extreme events. Nevertheless, climate models are essential tools for studying the dynamics of the most extreme events and for estimating future changes. Hence, given the increasing availability of SMILEs, we argue they should be preferred over single-ensemble model simulations to provide stakeholders with well-informed information on future climate risks associated with compound events.

## Methods

### Data

We used seven SMILEs: CESM1-CAM5^117^ (including 40 ensemble members), CSIRO-Mk3-6-0^118^ (30), CanESM2^90^ (50), EC-EARTH^119^ (16), GFDL-CM3^120^ (20), GFDL-ESM2M^121^ (30), and MPI-GE^122^ (100), providing data for the period 1950-2099 (based on the RCP8.5 emission scenario^123^ after 2005). We considered the period 1950–1980 as the historical baseline and periods of the same length in a world 2 °C (or 3 °C, in Fig. 6) warmer than pre-industrial conditions in 1870–1900^26^.

### Compound events

Given a variable and a model, extremes were defined as values exceeding a percentile-based threshold of the variable’s distribution obtained by pooling together data of the historical period from all available ensemble members of the model. Compound event frequencies were computed empirically via counting^26,53,94^.

Compound hot-dry events were defined as high mean temperature (above the 90th percentile) and low mean precipitation (below the 10th percentile) values over the warm season (the three consecutive months with highest mean temperature during 1950–1980)^26^. In Fig. 4, to quantify historical and future Spearman correlation between mean temperature and precipitation, for each ensemble member and grid point, we first removed linear trends in the two variables^53^ (detrending different ensemble members independently).

To study multi-year droughts, we defined droughts as annual average soil moisture over the total column below the 20th percentile (model MPI-GE). To assess concurrent droughts across soybean growing regions, we used the five soybean regions from Gaupp et al.^13^; here, a drought was defined when the regionally averaged annual soil moisture was below the 25th percentile. For droughts and compound hot-dry events, data were bilinearly interpolated to a 2.5° spatial grid before all calculations.

For concurrent wind and precipitation extremes, we considered historical daily data of accumulated precipitation and mean wind speed on the native model grid (model CESM1-CAM5). Frequencies of concurrent daily extremes (*f*_PW_) are based on precipitation and wind extremes defined as values occurring twice a year on average (i.e., values above their 100 ⋅ (1–2/365)th percentiles).

Overall, we note that considering more extreme thresholds to define compound events (e.g., the 16th percentile to define a soil moisture drought) would result in higher relative uncertainties in the frequency of compound events than those shown in Fig. 2.

### Ensemble mean and uncertainty quantification

Each of the seven SMILEs consists of several simulations starting from different initial conditions, resulting in multiple ensemble members that span a range of plausible climates and associated distributions for any quantity of interest *X*. Given a SMILE, we obtained robust estimates of *X* as the average of *X* from individual ensemble members (*ensemble mean*). Furthermore, when *X* is a projected change, e.g., the change in the frequency of compound events (Δ*f*), the forced response of *f* in the considered SMILE is the ensemble mean of Δ*f*.

For a given SMILE, following Maher et al. (2021)^25^ and Bevacqua et al. (2022)^26^, we quantified the uncertainty in the statistical quantity *X* in a single realisation due to internal climate variability as twice the standard deviation of *X* from individual ensemble members of a given SMILE model (2 ⋅ *U*_IV_). In Fig. 6a, b, where we consider multiple SMILE models to compute such an uncertainty due to internal climate variability, we obtain an average of *U*_IV_ across models as ${U}_{{{{{{{{\rm{IV}}}}}}}}}=\sqrt{\frac{1}{{N}^{mod}}\mathop{\sum }\limits_{s=1}^{{N}^{mod}}{\sigma }^{2}({X}_{s})}$, where *N*^*mod*^ = 7 is the number of models, and *σ*(*X*_*s*_) is the standard deviation of *X* from individual ensemble members of the model *s*. In Fig. 6a, b, we show the uncertainty in the future *f*_HD_ due to model-to-model differences (*U*_MD_) relative to the sum of *U*_MD_ and *U*_IV_. To this end, we quantified *U*_MD_ based on the standard deviations of the ensemble-mean of *f*_HD_ in the seven SMILEs^26^.

### Dependence of uncertainty on sample size

In Fig. 2, following the procedure of Bevacqua et al.^26^, we computed the ratio of twice the standard deviation to the mean of *f* (i.e., $2\cdot {U}_{{{{{{{{\rm{IV}}}}}}}}}/\overline{f}$) across *N*_ens_ = 12 ensemble members (for compound precipitation-wind extremes we use *N*_ens_ = 30) of various fixed sample size *N*^years^. The *N*_ens_ members were obtained via sampling data from pooled concatenated historical ensemble members of the SMILE considered to analyse the compound event of interest. When assessing (1) compound hot-dry events and (2) precipitation-wind extremes, we randomly sampled calendar years^54^ from the concatenated data. For assessing (3) droughts over, e.g., two consecutive years, we first modified each ensemble member simulation by creating a dataset with pairs of all two consecutive soil moisture annual values (we disregarded the first year of each ensemble member for which no previous soil moisture value exists). Then, we concatenated these modified ensemble members. Finally, we sampled the *N*_ens_ ensemble members taking pairs from the pooled concatenated data. To obtain dashed and dotted lines in Fig. 2a, we first computed the Spearman correlation between temperature and precipitation (based on all pooled ensemble members) and then selected the grid points with the 35% strongest and weakest coupling (i.e., strongest and weakest negative correlation coefficient), respectively.

### Required sample size for attribution

Extending the approach of Zscheischler and Lehner (2022)^43^, based on synthetic data of two variables *X* and *Y* with a given Pearson correlation cor_XY_, we quantified—in the presence of hypothetical combinations of anthropogenically forced positive trends Trend_X_ and Trend_Y_ in the two variables—the minimum sample size required for attribution of univariate high extremes and concurrent high extremes. We repeated the experiment three times, for different values of the correlations cor_XY_ (−0.5, 0, and +0.5). Note that we assume no trends in the compound drivers’ dependency^43^. Each experiment was carried out in three steps:

1. Based on a bivariate standard Gaussian distribution with a given correlation cor_XY_, we simulated *N* pairs (*X*, *Y*) both for a reference period representing hypothetical preindustrial conditions, and for a hypothetical present-day period under forced trends in the variables (via modifying the means of the distribution of the two variables by Trend_X_ and Trend_Y_). To identify the sample size *N* required for attribution, such simulations were conducted for a set of *N* ranging from 5 to 3000 and repeated *N*_bootstrap_ = 5000 times each.
2. For each of the *N*_bootstrap_ simulations associated with each sample size *N*, extreme events of individual variables were defined as values above the 90th percentile of the reference period. The probability ratio PR = *p*_1_/*p*_0_ was computed, where *p*_0_ is the empirically estimated probability of an extreme event occurring in the reference period simulations and *p*_1_ is that probability for the present-day period. PRs were computed for the two univariate extreme events and concurrent extremes.
3. We identified the minimum sample size *N* for which the attribution is *robust*, defined as a sample size for which the 95% confidence interval of PR is greater than one in 90% of the *N*_bootstrap_ simulations, where the confidence interval was estimated following Zscheischler and Lehner (2022)^42,43^.

### Event-based storylines

Compound events are found in a multidimensional space, for which, in contrast to a univariate space, there is no unique natural ordering, therefore identifying the most extreme compound events requires employing a metric whose extreme values are associated with meaningful events from an impact perspective. Here, we use a copula-based approach to select the most extreme events^77,78^. To identify extreme compound wintertime (December-February) precipitation and wind events, we first defined, for all pooled members of CESM1-CAM5, the variables *X*_1_ and *X*_2_ as the spatially-weighted average of daily precipitation and wind, respectively, over Portugal (Fig. 7). Secondly, through the R-package copula^124^, we computed the bivariate empirical copula C of these (*X*_1_, *X*_2_), that is the empirical joint cumulative distribution function^125^ of (*U*_1_ = F_1_(*X*_1_), *U*_2_ = F_2_(*X*_2_)), where F_*i*_ is the empirical cumulative distribution function of *X*_*i*_. The pairs (*X*_1_, *X*_2_) associated with the highest copula values (and therefore the highest probability Pr(*X*_1_ ≤ *x*_1_, *X*_2_ ≤ *x*_2_)) are then selected as the most extreme events. To identify extreme compound droughts across soybean breadbasket regions, we employed the same conceptual approach, but extended it to five dimensions and used data from the model MPI-GE. That is, we considered five variables (*X*_1_, *X*_2_, . . . , *X*_5_) that were defined as the negative values (given the interest in their extremely low values) of the breadbasket-regionally averaged annual soil moisture and computed the associated five-dimensional empirical copula C. Finally, the combinations (*X*_1_, …, *X*_5_) associated with the highest copula values were selected as the most extreme events.

### Perfect-model approach for evaluating a statistical model of spatially compounding droughts

For Fig. 8, we used the breadbasket regionally averaged annual soil moisture data for the historical period. We tested how a multivariate statistical model fitted to pseudo-observations with a limited sample size (here, 31 years from the first member) represents the compound event statistics of the underlying climate defined by pooling all available ensemble members (31 ⋅ 100 years).

Following the modelling of Bevacqua et al.^20^, the multivariate statistical model consists of a five-dimensional probability density function (pdf) of the variables (*X*_1_, *X*_2_, . . . , *X*_5_) (the soil moisture in the five breadbasket regions). The pdf is decomposed into the five marginal distributions of *X*_*i*_ and the copula density c, where c accounts for the dependence amongst the variables *X*_*i*_ regardless of their marginal distributions and was modelled via pair copula constructions (PCCs)^20^. Firstly, marginal distributions were fitted through a kernel density estimate^89^ (via the *stats* R-package^126^, function *density*). Then, we fitted pair-copula families and selected the best PCC structure via the function RVineStructureSelect (based on AIC) of the *VineCopula* R-package^127^. Copulas were fitted to the variables *U*_*i*_ = F_*i*_(*X*_*i*_), where F_*i*_ is the empirical cumulative distribution function of *X*_*i*_.

The resulting statistical model, i.e. the fitted 5-dimensional pdf, is associated with a unique probability of simultaneous droughts across N-regions, which is, however, affected by sampling uncertainty because the pdf is fitted to a sample of finite size. We estimated the sampling uncertainty via standard parametric bootstrap similar to previous studies^20,21,39^, in four steps. (1) We simulated *N*_*bootstrap*_ = 900 samples of (*X*_1_, *X*_2_, . . . , *X*_5_) with the same length as the original data (i.e., 31 years). This was achieved by simulating the variables (*u*_1_, *u*_2_, . . . , *u*_5_) and transforming them to (*x*_1_, *x*_2_, . . . , *x*_5_) via the inverse of the marginal kernel densities. (2) We fitted a pdf model to each of the *N*_*bootstrap*_ samples via the same procedure outlined above. (3) From each of these *N*_*bootstrap*_ models, we simulated a sample of (*X*_1_, *X*_2_, . . . , *X*_5_) with a length of 1000 times the original, such as to robustly obtain *N*_*bootstrap*_ probabilities of simultaneous droughts across N-regions associated with each pdf. (4) Finally, we computed the centred 95% confidence interval of such *N*_*bootstrap*_ probabilities, i.e. the final sampling uncertainty in the probability of simultaneous droughts across N-regions (shown via grey boxplots in Fig. 8 along with an overlaid black line indicating the mean of the *N*_*bootstrap*_ probabilities).

Finally, we note that a user may be interested in carrying out the analyses considering different ensemble members of a given SMILE as pseudo-observations (or different SMILEs to carry the overall analysis).

## Data Availability

The model data used in the study are available online at https://www.earthsystemgrid.org/dataset/ucar.cgd.ccsm4.CLIVAR_LE.html (for CanESM2, CESM-LE, CSIRO-Mk3-6-0, GFDL-ESM2M, and GFDL-CM3) and at https://esgf-data.dkrz.de/projects/mpi-ge/ (for MPI-GE). The HadCRUT5 data set can be found at https://www.metoffice.gov.uk/hadobs/hadcrut5/. All maps were obtained by using the *oce* R-package^128^.

## Code availability

All custom codes are direct implementations of standard methods and techniques, described in detail in Methods. The code used to prepare the figures in this paper is available from the corresponding author upon reasonable request.

## References

[1] Zscheischler J (2018). Future climate risk from compound events. Nat. Clim. Change.

[2] Bevacqua E (2021). Guidelines for studying diverse types of compound weather and climate events. Earth’s Future.

[3] Seneviratne, S. I. et al. in *Climate Change 2021: The Physical Science Basis. Contribution of Working Group I to the Sixth Assessment Report of the Intergovernmental Panel on Climate Change* 1513–1766 (Cambridge University Press, 2021).

[4] Zscheischler J (2020). A typology of compound weather and climate events. Nat. Rev. Earth Environ..

[5] Geirinhas JL (2021). Recent increasing frequency of compound summer drought and heatwaves in Southeast Brazil. Environ. Res. Lett..

[6] Hanchey A (2021). Notes from the field: deaths related to Hurricane Ida reported by media-nine states, August 29–September 9, 2021. Morb. Mortal. Wkly. Rep..

[7] Bastos A (2021). Vulnerability of European ecosystems to two compound dry and hot summers in 2018 and 2019. Earth Syst. Dyn..

[8] Hari V, Rakovec O, Markonis Y, Hanel M, Kumar R (2020). Increased future occurrences of the exceptional 2018–2019 Central European drought under global warming. Sci. Rep..

[9] Enqvist JP, Ziervogel G (2019). Water governance and justice in Cape Town: An overview. Wiley Interdiscip. Rev.: Water.

[10] Otto FE (2018). Anthropogenic influence on the drivers of the Western Cape drought 2015–2017. Environ. Res. Lett..

[11] Raymond C (2020). Understanding and managing connected extreme events. Nat. Clim. change.

[12] Singh J, Ashfaq M, Skinner CB, Anderson WB, Singh D (2021). Amplified risk of spatially compounding droughts during co-occurrences of modes of natural ocean variability. npj Clim. Atmos. Sci..

[13] Gaupp F, Hall J, Hochrainer-Stigler S, Dadson S (2020). Changing risks of simultaneous global breadbasket failure. Nat. Clim. Change.

[14] Gaupp F, Hall J, Mitchell D, Dadson S (2019). Increasing risks of multiple breadbasket failure under 1.5 and 2 ^*o*^C global warming. Agric. Syst..

[15] Kornhuber K (2020). Amplified Rossby waves enhance risk of concurrent heatwaves in major breadbasket regions. Nat. Clim. Change.

[16] Leonard M (2014). A compound event framework for understanding extreme impacts. Wiley Interdiscip. Rev.: Clim. Change.

[17] Zscheischler J, Sillmann J, Alexander L (2022). Introduction to the special issue: compound weather and climate events. Weather Clim. Extremes..

[18] Pescaroli G, Alexander D (2018). Understanding compound, interconnected, interacting, and cascading risks: a holistic framework. Risk Anal..

[19] Bellman, R., Corporation, R. & Collection, K. M. R. *Dynamic Programming*. *Rand Corporation Research Study* (Princeton University Press, 1957).

[20] Bevacqua E, Maraun D, Hobæk Haff I, Widmann M, Vrac M (2017). Multivariate statistical modelling of compound events via pair-copula constructions: analysis of floods in Ravenna (Italy). Hydrol. Earth Syst. Sci..

[21] Serinaldi F (2016). Can we tell more than we can know? The limits of bivariate drought analyses in the United States. Stoch. Environ. Res. Risk Assess..

[22] Eyring V (2016). Overview of the Coupled Model Intercomparison Project Phase 6 (CMIP6) experimental design and organization. Geosci. Model Dev..

[23] Hawkins E, Sutton R (2009). The potential to narrow uncertainty in regional climate predictions. Bull. Am. Meteorol. Soc..

[24] Deser C (2020). Insights from Earth system model initial-condition large ensembles and future prospects. Nat. Clim. Change.

[25] Maher N, Power SB, Marotzke J (2021). More accurate quantification of model-to-model agreement in externally forced climatic responses over the coming century. Nat. Commun..

[26] Bevacqua E, Zappa G, Lehner F, Zscheischler J (2022). Precipitation trends determine future occurrences of compound hot–dry events. Nat. Clim. Change.

[27] Maher N, Milinski S, Ludwig R (2021). Large ensemble climate model simulations: introduction, overview, and future prospects for utilising multiple types of large ensemble. Earth Syst. Dyn..

[28] Monerie P-A, Robson J, Dong B, Hodson D (2021). Role of the Atlantic multidecadal variability in modulating East Asian climate. Clim. Dyn..

[29] Seager R, Ting M (2017). Decadal drought variability over North America: mechanisms and predictability. Curr. Clim. Change Rep..

[30] Tavakol A, Rahmani V, Harrington Jr J (2020). Probability of compound climate extremes in a changing climate: A copula-based study of hot, dry, and windy events in the central United States. Environ. Res. Lett..

[31] Squire DT (2021). Likelihood of unprecedented drought and fire weather during Australia’s 2019 megafires. npj Clim. Atmos. Sci..

[32] Bevacqua E (2021). Larger spatial footprint of wintertime total precipitation extremes in a warmer climate. Geophys. Res. Lett..

[33] Wang R, Lü G, Ning L, Yuan L, Li L (2021). Likelihood of compound dry and hot extremes increased with stronger dependence during warm seasons. Atmos. Res..

[34] Hao Z (2020). Impact of dependence changes on the likelihood of hot extremes under drought conditions in the United States. J. Hydrol..

[35] Chen L, Chen X, Cheng L, Zhou P, Liu Z (2019). Compound hot droughts over China: Identification, risk patterns and variations. Atmos. Res..

[36] He X, Sheffield J (2020). Lagged compound occurrence of droughts and pluvials globally over the past seven decades. Geophys. Res. Lett..

[37] Cheng L (2016). How has human-induced climate change affected California drought risk?. J. Clim..

[38] Manning C (2019). Increased probability of compound long-duration dry and hot events in Europe during summer (1950–2013). Environ. Res. Lett..

[39] Ribeiro AFS, Russo A, Gouveia CM, Páscoa P, Zscheischler J (2020). Risk of crop failure due to compound dry and hot extremes estimated with nested copulas. Biogeosciences.

[40] Otto FE (2016). The attribution question. Nat. Clim. Change.

[41] Bindoff, N. L. et al. Detection and Attribution of Climate Change: from Global to Regional. In *Climate change 2013: The Physical Science Basis. Contribution of Working Group I to the Fifth Assessment Report of the Intergovernmental Panel on Climate Change* (Cambridge University Press, 2013).

[42] Paciorek CJ, Stone DA, Wehner MF (2018). Quantifying statistical uncertainty in the attribution of human influence on severe weather. Weather Clim. Extremes.

[43] Zscheischler J, Lehner F (2022). Attributing compound events to anthropogenic climate change. Bull. Am. Meteorol. Soc..

[44] Chiang F, Greve P, Mazdiyasni O, Wada Y, AghaKouchak A (2021). A multivariate conditional probability ratio framework for the detection and attribution of compound climate extremes. Geophys. Res. Lett..

[45] Kiriliouk A, Naveau P (2020). Climate extreme event attribution using multivariate peaks-over-thresholds modeling and counterfactual theory. Ann. Appl. Stat..

[46] Verschuur J, Li S, Wolski P, Otto FE (2021). Climate change as a driver of food insecurity in the 2007 Lesotho-South Africa drought. Sci. Rep..

[47] Massey N (2015). weather@ home-development and validation of a very large ensemble modelling system for probabilistic event attribution. Q. J. R. Meteorol. Soc..

[48] Robin Y, Ribes A (2020). Nonstationary extreme value analysis for event attribution combining climate models and observations. Adv. Stat. Climatol. Meteorol. Oceanogr..

[49] Deser C, Phillips A, Bourdette V, Teng H (2012). Uncertainty in climate change projections: the role of internal variability. Clim. Dyn..

[50] Matthews T, Wilby RL, Murphy C (2019). An emerging tropical cyclone–deadly heat compound hazard. Nat. Clim. Change.

[51] Deser C, Knutti R, Solomon S, Phillips AS (2012). Communication of the role of natural variability in future North American climate. Nat. Clim. Change.

[52] Wahl T, Jain S, Bender J, Meyers SD, Luther ME (2015). Increasing risk of compound flooding from storm surge and rainfall for major US cities. Nat. Clim. Change.

[53] Zscheischler J, Seneviratne SI (2017). Dependence of drivers affects risks associated with compound events. Sci. Adv..

[54] Bevacqua E (2020). More meteorological events that drive compound coastal flooding are projected under climate change. Commun. Earth Environ..

[55] Pendergrass AG, Knutti R, Lehner F, Deser C, Sanderson BM (2017). Precipitation variability increases in a warmer climate. Sci. Rep..

[56] Lesk C (2021). Stronger temperature–moisture couplings exacerbate the impact of climate warming on global crop yields. Nat. food.

[57] Chegwidden OS (2019). How do modeling decisions affect the spread among hydrologic climate change projections? Exploring a large ensemble of simulations across a diversity of hydroclimates. Earth’s Future.

[58] Bevacqua E, Zappa G, Shepherd TG (2020). Shorter cyclone clusters modulate changes in European wintertime precipitation extremes. Environ. Res. Lett..

[59] Simpson IR (2021). Emergent constraints on the large-scale atmospheric circulation and regional hydroclimate: do they still Work in CMIP6 and how much can they actually constrain the future?. J. Clim..

[60] Lehner F (2020). Partitioning climate projection uncertainty with multiple large ensembles and CMIP5/6. Earth Syst. Dyn..

[61] Zappa G, Shepherd TG (2017). Storylines of atmospheric circulation change for European regional climate impact assessment. J. Clim..

[62] Deser C (2020). Certain uncertainty: The role of internal climate variability in projections of regional climate change and risk management. Earth’s Future.

[63] Mankin JS, Lehner F, Coats S, McKinnon KA (2020). The value of initial condition large ensembles to robust adaptation decision-making. Earth’s Future.

[64] Lazenby MJ, Todd MC, Chadwick R, Wang Y (2018). Future precipitation projections over central and southern Africa and the adjacent Indian Ocean: What causes the changes and the uncertainty?. J. Clim..

[65] Sutton RT (2019). Climate science needs to take risk assessment much more seriously. Bull. Am. Meteorol. Soc..

[66] Sillmann J (2021). Event-based storylines to address climate risk. Earth’s Future.

[67] Shepherd TG (2018). Storylines: an alternative approach to representing uncertainty in physical aspects of climate change. Clim. Change.

[68] Hazeleger W (2015). Tales of future weather. Nat. Clim. Change.

[69] Meredith EP, Semenov VA, Maraun D, Park W, Chernokulsky AV (2015). Crucial role of Black Sea warming in amplifying the 2012 Krymsk precipitation extreme. Nat. Geosci..

[70] Schaller N (2020). The role of spatial and temporal model resolution in a flood event storyline approach in western Norway. Weather Clim. Extremes.

[71] Chan WC, Shepherd TG, Facer-Childs K, Darch G, Arnell NW (2022). Tracking the methodological evolution of climate change projections for UK river flows. Prog. Phys. Geogr. Earth Environ..

[72] Maraun D (2022). A severe landslide event in the Alpine foreland under possible future climate and land-use changes. Commun. Earth Environ..

[73] Fischer E, Sippel S, Knutti R (2021). Increasing probability of record-shattering climate extremes. Nat. Clim. Change.

[74] Thompson V (2017). High risk of unprecedented UK rainfall in the current climate. Nat. Commun..

[75] Gessner C, Fischer EM, Beyerle U, Knutti R (2021). Very rare heat extremes: quantifying and understanding using ensemble reinitialization. J. Clim..

[76] van der Wiel K, Lenderink G, de Vries H (2021). Physical storylines of future European drought events like 2018 based on ensemble climate modelling. Weather Clim. Extremes.

[77] Brunner MI, Gilleland E, Wood AW (2021). Space–time dependence of compound hot–dry events in the United States: assessment using a multi-site multi-variable weather generator. Earth Syst. Dyn..

[78] Li J (2022). Regional asymmetry in the response of global vegetation growth to springtime compound climate events. Commun. Earth Environ..

[79] Hénin R, Ramos AM, Pinto JG, Liberato ML (2021). A ranking of concurrent precipitation and wind events for the Iberian Peninsula. Int. J. Climatol..

[80] Kelder T (2022). Interpreting extreme climate impacts from large ensemble simulations-are they unseen or unrealistic?. Environ. Res. Lett..

[81] Tilloy A, Malamud BD, Winter H, Joly-Laugel A (2019). A review of quantification methodologies for multi-hazard interrelationships. Earth Sci. Rev..

[82] Jane R, Cadavid L, Obeysekera J, Wahl T (2020). Multivariate statistical modelling of the drivers of compound flood events in south Florida. Nat. Hazards Earth Syst. Sci..

[83] Huang WK, Monahan AH, Zwiers FW (2021). Estimating concurrent climate extremes: A conditional approach. Weather Clim. Extremes.

[84] Vignotto E, Engelke S, Zscheischler J (2021). Clustering bivariate dependencies of compound precipitation and wind extremes over Great Britain and Ireland. Weather Clim. Extremes.

[85] Vogel J (2021). Identifying meteorological drivers of extreme impacts: an application to simulated crop yields. Earth Syst. Dyn..

[86] Zscheischler J, Naveau P, Martius O, Engelke S, Raible CC (2021). Evaluating the dependence structure of compound precipitation and wind speed extremes. Earth Syst. Dyn..

[87] Engelke S, Ivanovs J (2021). Sparse structures for multivariate extremes. Annu. Rev. Stat. Appl..

[88] Boulaguiem Y, Zscheischler J, Vignotto E, van der Wiel K, Engelke S (2022). Modeling and simulating spatial extremes by combining extreme value theory with generative adversarial networks. Environ. Data Sci..

[89] Manning C (2018). Soil moisture drought in Europe: a compound event of precipitation and potential evapotranspiration on multiple time scales. J. Hydrometeorol..

[90] Kirchmeier-Young MC, Zwiers FW, Gillett NP (2017). Attribution of extreme events in Arctic sea ice extent. J. Clim..

[91] Schaller N (2018). Influence of blocking on Northern European and Western Russian heatwaves in large climate model ensembles. Environ. Res. Lett..

[92] Singh H, Najafi MR, Cannon AJ (2021). Characterizing non-stationary compound extreme events in a changing climate based on large-ensemble climate simulations. Clim. Dyn..

[93] Poschlod B, Zscheischler J, Sillmann J, Wood RR, Ludwig R (2020). Climate change effects on hydrometeorological compound events over southern Norway. Weather Clim. Extremes.

[94] Raymond C (2022). Increasing spatiotemporal proximity of heat and precipitation extremes in a warming world quantified by a large model ensemble. Environ. Res. Lett..

[95] Touma D (2022). Climate change increases risk of extreme rainfall following wildfire in the western United States. Sci. Adv..

[96] Bartusek S, Kornhuber K, Ting M (2022). 2021 North American heatwave amplified by climate change-driven nonlinear interactions. Nat. Clim. Change.

[97] Berg A (2015). Interannual coupling between summertime surface temperature and precipitation over land: Processes and implications for climate change. J. Clim..

[98] Priestley MD, Catto JL (2022). Future changes in the extratropical storm tracks and cyclone intensity, wind speed, and structure. Weather Clim. Dyn..

[99] Villalobos-Herrera R (2021). Towards a compound-event-oriented climate model evaluation: a decomposition of the underlying biases in multivariate fire and heat stress hazards. Nat. Hazards Earth Syst. Sci..

[100] Suarez-Gutierrez L, Milinski S, Maher N (2021). Exploiting large ensembles for a better yet simpler climate model evaluation. Clim. Dyn..

[101] Owen LE, Catto JL, Dunstone NJ, Stephenson DB (2021). How well can a seasonal forecast system represent three hourly compound wind and precipitation extremes over Europe?. Environ. Res. Lett..

[102] Maraun D (2017). Towards process-informed bias correction of climate change simulations. Nat. Clim. Change.

[103] Switanek M, Maraun D, Bevacqua E (2022). Stochastic downscaling of gridded precipitation to spatially coherent subgrid precipitation fields using a transformed Gaussian model. Int. J. Climatol..

[104] François B, Vrac M, Cannon AJ, Robin Y, Allard D (2020). Multivariate bias corrections of climate simulations: which benefits for which losses?. Earth Syst. Dyn..

[105] Vrac M (2018). Multivariate bias adjustment of high-dimensional climate simulations: the Rank Resampling for Distributions and Dependences (R 2 D 2) bias correction. Hydrol. Earth Syst. Sci..

[106] Cannon AJ (2018). Multivariate quantile mapping bias correction: an N-dimensional probability density function transform for climate model simulations of multiple variables. Clim. Dyn..

[107] Robin Y, Vrac M, Naveau P, Yiou P (2019). Multivariate stochastic bias corrections with optimal transport. Hydrol. Earth Syst. Sci..

[108] Zscheischler J, Fischer EM, Lange S (2019). The effect of univariate bias adjustment on multivariate hazard estimates. Earth Syst. Dyn..

[109] Vaittinada Ayar P, Vrac M, Mailhot A (2021). Ensemble bias correction of climate simulations: preserving internal variability. Sci. Rep..

[110] Cannon, A. J., Alford, H., Shrestha, R. R., Kirchmeier-Young, M. C. & Najafi, M. R. Canadian Large Ensembles Adjusted Dataset version 1 (CanLEADv1): Multivariate bias-corrected climate model outputs for terrestrial modelling and attribution studies in North America. *R. Meteorol. Soc*. 10.1002/gdj3.142 (2021).

[111] Warszawski L (2014). The inter-sectoral impact model intercomparison project (ISI–MIP): project framework. Proc. Natl Acad. Sci. USA.

[112] van der Wiel K (2019). The influence of weather regimes on European renewable energy production and demand. Environ. Res. Lett..

[113] McKinnon KA, Deser C (2018). Internal variability and regional climate trends in an observational large ensemble. J. Clim..

[114] Beusch L (2022). From emission scenarios to spatially resolved projections with a chain of computationally efficient emulators: coupling of MAGICC (v7. 5.1) and MESMER (v0. 8.3). Geosci. Model Dev..

[115] Deser C, Terray L, Phillips AS (2016). Forced and internal components of winter air temperature trends over North America during the past 50 years: Mechanisms and implications. J. Clim..

[116] Sippel S (2019). Uncovering the forced climate response from a single ensemble member using statistical learning. J. Clim..

[117] Kay JE (2015). The Community Earth System Model (CESM) large ensemble project: A community resource for studying climate change in the presence of internal climate variability. Bull. Am. Meteorol. Soc..

[118] Jeffrey S (2013). Australia’s CMIP5 submission using the CSIRO-Mk3.6 model. Aust. Meteor. Oceanogr. J..

[119] Hazeleger W (2010). EC-Earth: A seamless Earth-system prediction approach in action. Bull. Am. Meteorol. Soc..

[120] Sun L, Alexander M, Deser C (2018). Evolution of the global coupled climate response to Arctic sea ice loss during 1990–2090 and its contribution to climate change. J. Clim..

[121] Rodgers KB, Lin J, Frölicher TL (2015). Emergence of multiple ocean ecosystem drivers in a large ensemble suite with an Earth system model. Biogeosciences.

[122] Maher N (2019). The Max Planck Institute Grand Ensemble: enabling the exploration of climate system variability. J. Adv. Model. Earth Syst..

[123] Moss RH (2010). The next generation of scenarios for climate change research and assessment. Nature.

[124] Kojadinovic I, Yan J (2010). Modeling multivariate distributions with continuous margins using the copula R package. J. Stat. Softw..

[125] Sklar M (1959). Fonctions de repartition an dimensions et leurs marges. Publ. inst. Stat. Univ. Paris.

[126] R Core Team. *R: A Language and Environment for Statistical Computing*. (R Foundation for Statistical Computing, 2013).

[127] Schepsmeier, U. et al. *VineCopula: Statistical Inference of Vine Copulas*. https://rdrr.io/cran/VineCopula/man/VineCopula-package.html (2016).

[128] Kelley, D., Richards, C. & Layton, C. *oce: Analysis of Oceanographic Data*. https://cran.r-project.org/web/packages/oce/oce.pdf (2022).

